# Longer Nature-Based Rehabilitation May Contribute to a Faster Return to Work in Patients with Reactions to Severe Stress and/or Depression

**DOI:** 10.3390/ijerph14111310

**Published:** 2017-10-27

**Authors:** Patrik Grahn, Anna María Pálsdóttir, Johan Ottosson, Ingibjörg H. Jonsdottir

**Affiliations:** 1 Department of Work Science, Business Economics and Environmental Psychology, Swedish University of Agricultural Sciences, P.O. Box 88, SE-230 53 Alnarp, Sweden; anna.maria.palsdottir@slu.se (A.M.P.); johan@strandhusen.se (J.O.); 2 The Institute of Stress Medicine, Region Västra Götaland, Carl Skottsbergs Gata 22B, SE 413 19 Gothenburg, Sweden; ingibjorg.jonsdottir@vgregion.se

**Keywords:** horticultural therapy, burnout, vocational therapy, common mental disorders, dose respond, healing garden

## Abstract

The global burden of depression and stress-related mental disorders is substantial, and constitutes a major need for effective rehabilitation. Can nature-based rehabilitation help people return to work? Objective: To study if the length of a nature-based rehabilitation program affects the outcome with regard to return to work one year after the onset of the program, in a group of patients with long-term reactions to severe stress and/or depression. Methods: A prospective, quasi-experimental study comparing results from 8-, 12-, and 24-week periods of rehabilitation. The rehabilitation of 106 participants was carried out by a multimodal rehabilitation team in a specially designed rehabilitation garden. Return to work data were collected before the intervention and one year after the start of rehabilitation. In addition, data were collected regarding self-assessed occupational competence, personal control, and sense of coherence. As many as 68% of the participants returned to work or participated in job training or work-oriented measures, full- or part-time, after one year. Participants with a longer period of rehabilitation reported better results on occupational competence, and were more likely to participate in paid work, full-time or part-time, one year after rehabilitation. Study outcomes indicate that a longer rehabilitation period in a rehabilitation garden increases the possibility of a return to paid work.

## 1. Introduction

The global burden of mental disorders is substantial [1,2]. In Sweden, the contribution of psychiatric disorders to workplace absenteeism has increased markedly since 2007, and now constitutes the majority of long-term illnesses for both men and women [3]. Psychosocial factors at work are believed to be one of the main contributors to this increase [4]. Consequently, knowledge with regard to both the prevention and rehabilitation of stress-related mental health problems is urgent. In a review of the effects of rehabilitation on workplace absenteeism, work ability, and return to work, Kuoppala and Lamminpää [5] point out that the scientific support for rehabilitation is generally weak. There is moderate evidence that multimodal rehabilitation increases the possibility of return to work, whereas counseling, exercise, and return-to-work programs do not have any significant effect on return to work rates at one year after the start of rehabilitation. This is supported by an extensive review [6] concerning return to work after mild psychiatric illness. However, there is limited scientific support that return to work rates increase if the workplace is involved in the rehabilitation [7,8]. As such, the increasing amounts of absenteeism attributed to stress- and work-related factors underline the need for effective rehabilitation programs.

There is increasing scientific evidence that the natural environment can relieve symptoms of stress and improve recovery from mental disorders [9,10,11,12,13,14]. Nature-based interventions [15] may therefore be appropriate for people who are absent from work due to stress-related mental disorders. Different diagnoses are used to define this group of patients depending on the symptoms. Patients on sick-leave due to stress-related mental health problems most commonly are diagnosed with reaction to severe stress and/or depression [16].

There is a long tradition of the use of nature-based therapy and rehabilitation in healthcare; horticultural therapy (HT) is one example [17]. Since the late 1990s, research in this area has progressively increased, particularly as regards the elderly and patients with mental disorders. Six extensive reviews [18,19,20,21,22,23] conclude that nature-based rehabilitation, such as HT, leads to improvements in different outcomes for various diagnoses. The effect size seems to be highest for diagnoses related to mild to moderate mental illness [22,23].

There are a number of theories available that offer an explanation of why nature-based interventions are good for mental health. For a long time, the health effects of staying in natural landscapes and urban parks were linked to daylight, fresh air, and physical activity. Such theories are still current [15]. Attention Restoration Theory (ART) [24] claims that people possess two different types of attention. Directed attention is a type of attention steered by one’s intention, which means that the individual needs to focus his/her attention to be able to solve a task. The other type of attention, fascination, is involuntary attention, which means that the individual spontaneously pays attention to something. This can range from a soft glitter in a water surface to a sudden hooting horn from a car. People have a limited capacity of directed attention; it needs to be restored so that they can carry through both work and everyday life. Directed attention is a fundamental part of our executive function [25,26]. Using fascination does not leave you fatigued. On the contrary: the theory claims that nature has a wide range of attributes that attract soft fascination, such as chirping birds or the sound of the breeze in the tree-crowns: impressions you can rest in. This gives room for restoration of directed attention [24]. The Psycho-Evolutionary Theory (PET) [27] claims that the human being, through evolution, has always been exposed to stress. Humans have therefore been equipped with the ability to assess very quickly whether a natural environment is dangerous or not. Dark forests, high precipices, or the presence of large predators or snakes raise the stress level. Being in serene, bright open natural environments, especially when having a prospect over water surfaces, reduces stress. In natural environments this mechanism works, because humans through evolution are adapted to natural landscapes, but this mechanism does not work indoors or in cities without greenery. People who regularly visit natural environments can therefore recover quickly from high stress levels [27].

The Supportive Environment Theory (SET) [28] is a biopsychosocial theory [29] based on the assumption that humans have evolved in such a way as to be adapted to a life close to nature, which involves social and cultural interaction with a limited number of people. This theory is specifically designed for nature-based interventions [15,30,31,32]. “Supportive environments” consist of physical (e.g., natural and built environments), social (e.g., friends, colleagues, and family), and cultural environments (e.g., work, leisure, and values). SET claims that people need supportive environments in order to develop physically (senses, muscles, and locomotion) and mentally (the ability to feel and think) in close interaction with the physical, social, and cultural environment [28]. Nature-based interventions are therefore designed to provide a supportive physical environment (especially selected or specially designed), supportive activities, and a supportive social environment, where the intervention is often conducted by a multimodal team [15,33]. If participants can stay in a supportive natural environment, they are expected to recover from high stress levels and low capabilities regarding directed attention, as assumed by the PET and ART. In addition, muscle strength and general physical fitness are assumed to increase [28,33]. Moreover, through group activities in a supportive environment, coping capability is assumed to increase, as well as sense of coherence and executive functions [28,31,33,34]. The improvement is expected to take place in several stages, starting with being able to go to the rehabilitation center and stay there for a couple of hours [30,31,32]. After some weeks, the participants can start vocational rehabilitation and at length return to work [28,33,34]. The theory focuses primarily on diagnoses related to stress-related mental illness [28]. When it comes to people suffering from severe stress-related illness, it is known that it takes a long time to return to work [16]. Ordinary multimodal rehabilitation programs are eight, twelve, or twenty-four weeks in Sweden. SET assumes that a longer time in a nature-based intervention gives more effect, but the amount of time required is open [28,33].

A number of nature-based interventions have been designed specifically for the rehabilitation of stress-related mental disorders and/or depression, and the results have generally been shown to be good concerning outcomes related to stress-reduction, the ability to focus attention [35,36,37,38], or healthcare consumption [39]. Some studies, however, indicate mixed results: certain values improve (e.g., symptom reduction), while others do not (e.g., quality of life) [40,41]. Very few studies have been conducted measuring return to work [36]; thus, there is a gap in knowledge.

The Alnarp Rehabilitation Garden at the Swedish University of Agricultural Sciences (SLU) campus outside Malmö, in southern Sweden, has been specially designed to offer rehabilitation programs for people with stress-related psychiatric disorders and/or depression [33]. A significant reduction in healthcare consumption was noted among participants treated at Alnarp one year after they had received nature-based vocational rehabilitation when compared with a matched population-based reference cohort [39]. This was a retrospective cohort study, with a matched reference group from the general population. Each participant who was referred to the nature-based rehabilitation program was matched with seven controls recruited from the county council health care register. For both groups, information on healthcare consumption was extracted from the county council health care register.

### Hypotheses and Objectives

The program at the Alnarp Rehabilitation Garden was designed according to the Supportive Environment Theory [28]. Our main hypothesis, which is derived from this theory, was that longer rehabilitation would increase the likelihood of returning to work among patients suffering from stress-related mental disorders. According to the theory, this would be due to improvements in executive function, sense of personal control, and sense of coherence.

Thus, the main aim of this study was to examine return-to-work rates one year after participating in a rehabilitation program with different durations at the Alnarp Rehabilitation Garden. In addition, we examined self-assessed occupational competence, personal control, and sense of coherence before and after nature-based vocational rehabilitation.

## 2. Materials and Methods

### 2.1. Study Population

The Alnarp Rehabilitation Garden has a framework agreement with the Swedish Social Insurance Agency stipulating a fixed cost per treatment day. This agreement made it possible to offer three different lengths of rehabilitation programs: 8 weeks, 12 weeks, and 24 weeks. If any participant needed additional psychotherapy or physical therapy after this time, this could be arranged at the Rehabilitation Garden. Since this type of intervention is defined as vocational rehabilitation, decisions on program length were made by the social insurance officers who recommended patients for the program, rather than those in the health care system who were otherwise responsible for the patients’ medical treatment. A social insurance officer is an official who manages the payment of sickness compensation from the Swedish Social Insurance Agency. The official is not medically trained. Some officials decided that their patients should receive 8 weeks of rehabilitation in the garden, while others opted for periods of 12 or 24 weeks. The duration of the program was not determined by the level of sickness of the participants. Everyone was considered healthy enough to participate in a vocational rehabilitation. The rehabilitation program was provided in addition to any possible ongoing treatment with medication.

The social insurance officers referred patients who were on long-term sick leave and had a diagnosis of reaction to severe stress and/or a depressive episode. Each participant took part in rehabilitation for the amount of time ordered. No randomization was performed. This type of study can be compared to a natural experiment [42,43]. The scientific approach we used in this study can also be described as quasi-experimental [44,45,46] with intention to treat [47], although the population was not randomized in the usual way.

Participants who met the following inclusion and exclusion criteria were considered eligible for the study, and could be referred through the framework agreement: (1) being on sick leave for at least three months due to a diagnosis of reaction to severe stress and/or a depressive episode, defined as having an International Statistical Classification of Diseases [48] within the F43 and/or F32 category; (2) good understanding of spoken and written Swedish; and (3) could not be suffering from any drug or alcohol abuse.

A total of 118 participants were referred to the rehabilitation program. All participants were on 100% sick leave. Most of them (87 participants) had not been able to work for more than two years. Before entering the rehabilitation program, the participants received written and oral information about the study, and had an opportunity to ask questions about the study procedure before signing informed consent. The participants could withdraw from the study at any point without this influencing their relationship with their therapist. The study was approved by the Research Ethics Committee at the Medical Faculty of Lund University (LU 107-02 and EPN Lund 2011/31).

**Figure 1:**
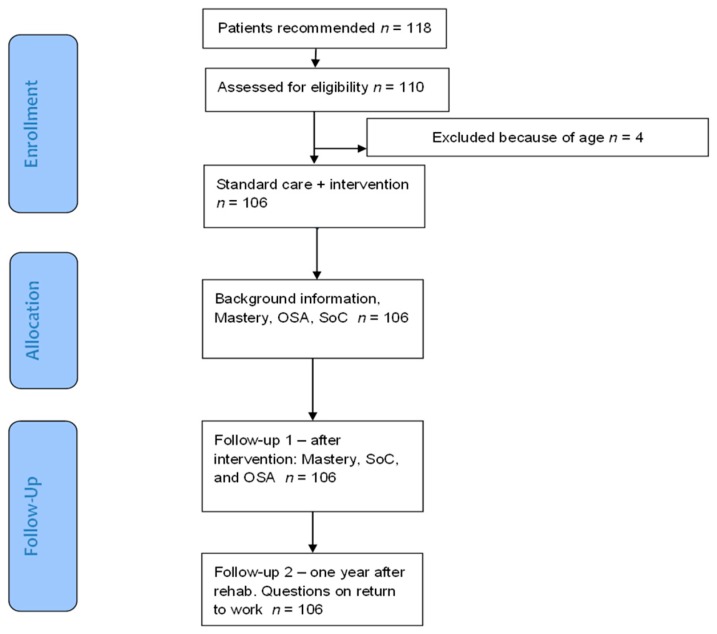
Flowchart. OSA: Occupational Self-Assessment; SoC: Sense of Coherence.

A psychiatrist who is an expert in stress-related psychiatric disorders and works part time in the Alnarp Rehabilitation Garden Program examined all participants in the rehabilitation program in order to reconsider or confirm their diagnoses before they were included in the study (Figure 1). This was done after two weeks of rehabilitation. Reaction to severe stress was the primary diagnosis for 79 of the participants (ICD-10 main condition code F43.8 or F43.9), while 31 participants were primarily suffering from a mild to moderate depressive episode (main condition code F32.0 or F32.1). Eight participants did not fulfill the inclusion criteria; five of these were diagnosed with chronic non-malignant pain (M79), two had whiplash (S13), and one had chronic fatigue syndrome after a viral infection (G93).

Of the 110 participants who fulfilled the inclusion criteria, 4 were excluded from further analysis and comparisons since they exceeded retirement age (65 years) prior to the 1-year follow-up, and it was therefore not possible to analyze them in terms of return to work. This left us with a total of 106 participants (96 women/10 men). Participants who were excluded from the study could continue the rehabilitation in the garden even though they were not included in the study.

Participants were aged 45.7 years on average (range 22–63), and had been suffering from their disorder for an average of 3 years and 7 months (CI 3.3–4.1 years). Sixty-four percent were mid-level officials who had received education at the university-level or equivalent, such as teachers and nurses, while eleven percent were high-ranking officials or employees with even greater levels of academic education, such as medical doctors and lawyers.

### 2.2. Procedure

The study was conducted in the following steps (Figure 1): all participants were asked to fill out questionnaires regarding background information, such as profession and family situation. In addition, all participants were asked to fill out protocols regarding work participation, occupational competence, personal control, and sense of coherence. 

All participants were followed during the period prior to the one-year follow-up. This included meetings at Alnarp every three months. At the final meeting, the participants were asked to answer a set of questions concerning their rehabilitation. All self-assessment questionnaires were filled out one week before the start of the intervention and one week after its completion, with the exception of the return-to-work questionnaire, which participants answered one year after the start of rehabilitation. All participants returned the questionnaires relating to background issues.

Questions during final meeting: Some of these questions related to the participants’ possible needs for additional rehabilitation, such as consultations with psychotherapists or physical therapists, before the one-year follow-up. Twenty-one of the participants sought and received additional rehabilitation. All of these additional consultations took place at the Alnarp Rehabilitation Garden. For two of the participants, this rehabilitation involved psychotherapy only, and for three it involved both psychotherapy and physical therapy. All five psychotherapy consultations involved a single visit to the psychotherapist. Sixteen participants took part in physical therapy only. There were usually five visits to the physical therapist before the one-year follow-up (range 1–12).

Table 1 shows the baseline characteristics of the participants broken down by rehabilitation period. Apart from the length of rehabilitation, there are no significant differences between the three groups.

### 2.3. The Rehabilitation Program

**Figure 2:**
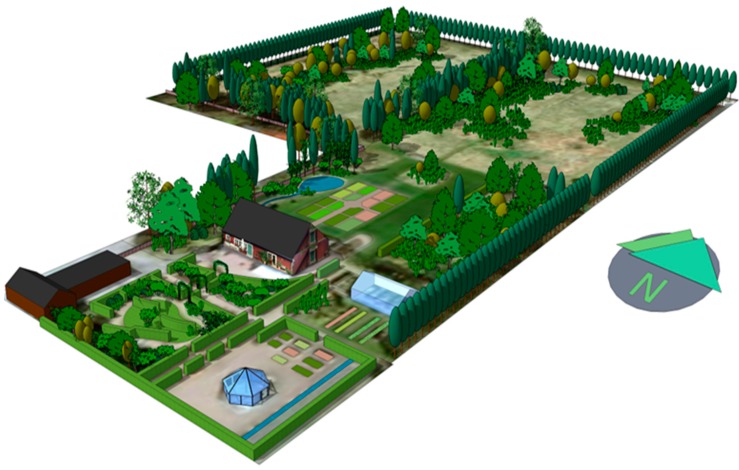
Alnarp Rehabilitation Garden, three-dimensional map made by Gunnar Cerwén.

The Alnarp Rehabilitation Garden is a clinical entity within the Swedish University of Agricultural Sciences (SLU) that is dedicated to the research and development of nature-based rehabilitation interventions. This clinical care unit only accepts patients participating in SLU-related research projects. The 2-hectare garden (Figure 2) is located on the SLU Alnarp campus in southern Sweden [33]. At the Alnarp Rehabilitation Garden, the Supportive Environment Theory is fully applied, providing support to the individual in several ways: a thoughtfully designed garden (support from the physical environment); meaningful activities (support from the cultural environment); and group rehabilitation with other participants in the same situation (support from the social environment). Support is also provided by a professional multimodal team: a registered occupational therapist; a registered psychotherapist; a registered physical therapist; a registered psychiatrist; a landscape architect; and a landscape engineer [28,34,49].

The garden design is based on the different theories mentioned above (ART, PET, and SET), as well as on earlier environmental psychological theories, such as the theory of Savannah-like environments [50], the Prospect-Refuge Theory [51], and theories on human preferences for water mirrors, lakes, and seas [52]. The design has also been based on qualities related to the perceived restorativeness scale [53], as well as qualities related to perceived sensory dimensions [54]. A more complete description of the design process can be found in [15,28,33,49].

A psychiatrist met with the participants in connection with program registration, again after a couple of weeks’ participation, and at the end of the intervention. An individual rehabilitation plan was designed for each participant. Rehabilitation took place 4 days a week—Monday through Thursday—for 3½ hours per day. The treatment was based on gardening and horticulture activities, with the occupational therapist and the landscape engineer supervising participants. Activities were changed with regard to season and weather, and we have not found any noticeable differences regarding the effects of the treatment in relation to the seasons [32]. Each rehabilitation group consisted of eight participants. The gardening and horticulture activities also involved resting in the garden or walking. The more work-oriented activities increased gradually, depending on the participant’s physical and mental condition. On Tuesday and Wednesday, participants were offered individual physical therapy and psychotherapy. Physical therapy was carried out in the garden whenever possible. Psychotherapy was performed either in a greenhouse, or while the psychotherapist and participant strolled in the garden or worked together [28]. A more complete description of the program can be found in Grahn et al. [28].

### 2.4. Outcome Measures

#### 2.4.1. Primary Outcome Measure

Self-rated return to work (RTW). The participants were asked to complete a form regarding their work situation at the start, and one year after the start, of rehabilitation. A questionnaire was mailed to them, and was followed up with two reminders. This questionnaire was designed at SLU Alnarp, and has been used previously by Palsdottir et al. [36]. The social insurance system in Sweden applies a system of 100%, 75%, 50%, and 25% with regard to work participation.

The question read: “What was your work situation on xx? (xx being one year from the date he/she started rehabilitation). As of this date, it is exactly one year since you started the rehabilitation program at Alnarp”.

The participants were asked to answer according to their actual work situation in percent of full time (100%, 75%, 50%, 25%, 0%, or other namely …%,) on the given date. The grades of occupational activities were further divided into the following categories:

- Paid work: “Paid work as employee”, “Self-employed”, “Actively applying for paid work as employee”, or “Student/studying”.
- Work training: “Vocational training in the workplace”.
- Vocational action: “Work-oriented measures by (or supported by) the Swedish Social Insurance Agency”.
- Still sick: “Sick-listed”.

We used paid work as the outcome variable to compare with other variables (for example, sense of coherence), since paid work sets higher demands than other forms of occupation.

#### 2.4.2. Secondary Outcome Measures

Occupational competence: The Occupational Self-Assessment protocol (OSA) derives from the model of human occupation (MOHO), which has become standard among occupational therapists ever since it was first presented in 1980 [55]. It is a client-centered instrument with 35 items that assess occupational competence. In an international cross-cultural study, OSA proved to be valid and reliable [56]. The protocol (21 items), addresses the ability to handle everyday problems and asks respondents to evaluate their competence in a number of areas, such as physical ability: “To be physically capable of doing what I need to do”; the ability to cope with social situations: “Expressing myself to others” and “Getting along with others”; as well as executive function: “Concentrating on my tasks”; “Identifying and solving problems”; “Making decisions based on what I think is important”; and “Accomplishing what I set out to do”.

Personal control: The Mastery Scale measures how participants experience their personal control, or how they master situations in everyday life [57,58]. The Mastery Scale consists of seven items that are answered on a four-point scale. The Mastery Scale is a reliable, valid, cross-culturally applicable instrument for measuring how people experience their personal control [59,60]. According to Stephens et al. [61] and Nyqvist et al. [62], the cut-off value is <23.

Sense of coherence: The original version of the Sense of Coherence scale (SoC-29) was used in this study. SoC-29 is considered to be reliable and valid [63]. According to Ahola et al. [64], the cut-off value is <63.

### 2.5. Statistical Analyses

To analyze variations regarding return to work rates between different rehabilitation programs, we used the Mantel–Haenszel Chi-Square test. To analyze changes regarding return to work and self-rated occupational competence, personal control, and sense of coherence before and after nature-based rehabilitation, a paired *t*-test analysis was used.

To analyze how different factors (background issues, length of intervention, changes in self-rated assessments) affected the outcome of the intervention with regard to return to work, we used a stepwise binary logistic regression analysis to find the best explanatory model. Variables which in a bivariate Spearman correlation analysis were associated with return to work, with *p*-values < 0.20, were inserted into the logistic regression analysis [65,66]. In the stepwise analysis, the Akaike information criterion was used to select the best model along with the likelihood ratio test and the Wald test. The Hosmer–Lemeshow test was used to ensure that the model showed no evidence of lack of fit. SAS software (release 9.2, Cary, NC, USA) was used for analysis, and a significance level of 0.05 was used.

We calculated the effect size [67] based on:

- differences between means, using Hedges’ g [68], where magnitude >0.80 equals high; >0.50 moderate; and >0.20 small.
- degree of association between binary variables, using odds ratio (OR) [69], where magnitude >3 equals high; >2 moderate; and >1.5 low.

## 3. Results

### 3.1. Return to Work Rates 12 Months After Rehabilitation

Forty-two participants (44%) had returned to full-time or part-time paid work after one year (Table 2). Of these, 14 participants had returned to full-time work.

Fifty-three participants had not returned to paid work. Of these, 23 participants took part in some kind of job training in a workplace or other forms of work-oriented measures, such as customized job training. Thirty participants did not participate in any work-related activity one year after the intervention (Table 2). After one year, however, 68.4% started to work or participated in job training or work-oriented measures on a full- or part-time basis. We lack information for 11 participants.

### 3.2. Associations Between Length of Rehabilitation Period and Return to Paid Work

**Figure 3:**
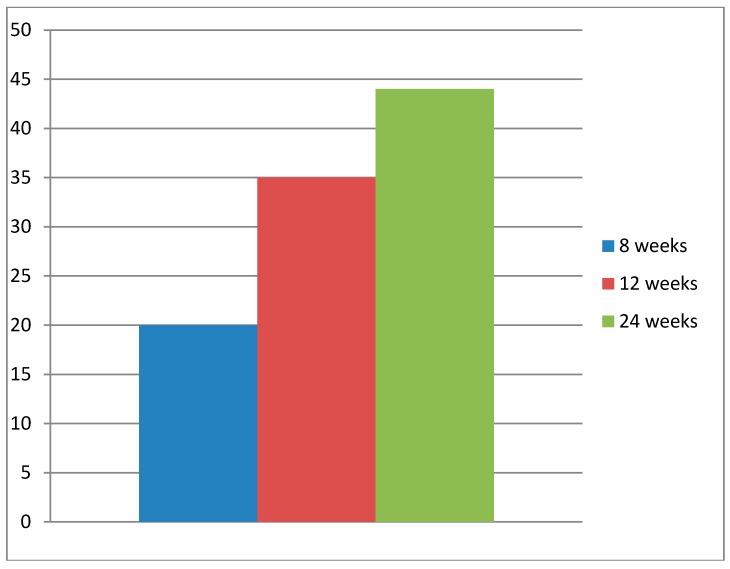
Percent paid work (*y*-axis), mean values, one year after start of rehabilitation, for the patients participating in the different rehabilitation periods.

Patients participating in the 24-week program reported a significantly higher (*p* < 0.05) percentage of full-time work (44%) one year after beginning the rehabilitation period compared to those participating in the 12-week (37%) and 8-week (20%) programs (Figure 3). The proportion that had returned to work after 12 weeks of rehabilitation was also significantly higher compared to those participating in the 8-week program (*p* < 0.05).

A comparison of participants enrolled in the 8-, 12-, and 24-week rehabilitation programs is illustrated in Table 3. Participants in the different programs demonstrated no significant differences in mean scores at baseline: e.g., the OSA score was approximately 36. Post-rehabilitation, however, noticeable differences were found. For all three programs, values concerning RTW, OSA, Mastery, and SoC improved significantly at the group level (Table 3). The effect size, as measured by Hedges’ g, increased with the number of weeks in the program, in terms of RTW and OSA, and was most powerful for RTW.

In the correlation analysis, we used the cut-off values for Mastery and the SoC protocol. We dichotomized OSA, age, and time on sick-leave at the mean. Socioeconomic status was also dichotomized, with manual worker, professional worker, and subordinate official/employee forming one group, and mid-level official/employee and high-ranking official/employee forming the other. Return to work was dichotomized at more than or equal to half-time paid work, and gender was already dichotomized. Variables associated with return to work in the correlation analysis with *p*-values < 0.20 were included in the stepwise regression analysis: marital status, socioeconomic status, length of rehabilitation period, OSA, and Cut-off SoC.

The analysis yielded a significant model (*p* < 0.01) including length of intervention (*p* < 0.01, OR 3.13) and OSA (ns, OR 2.24). The Hosmer and Lemeshow Test showed that there was no evidence of a lack of fit in any of these models.

## 4. Discussion

The main aim of this study was to examine return to work a year after the start of participation in nature-based rehabilitation programs with different lengths (8, 12, and 24 weeks). According to our main hypothesis, which is derived from the Supportive Environment Theory (SET), a longer nature-based rehabilitation program would increase the ability to return to work. This, in turn, would be due to improvements in occupational competence, personal control, and sense of coherence.

### 4.1. Return to Work

Our results show that forty-four percent of the participants had returned to paid work, most of them part-time, and just under 15% were able to go back to full-time paid work. An additional 24% participated in some kind of job training or customized job training on a full-time or part-time basis one year after the nature-based rehabilitation program. Thus, a total of 68% of the entire group were back to some work-place-related activities. It should be noted that the participants had not worked or studied for an average of 3 years and 7 months.

The effect of different intervention time periods was examined, and it was found that the longer the rehabilitation period, the higher the rate of return to the labor market. In the regression analysis, the length of the rehabilitation program was found to be the significant and decisive factor in predicting return to work. Sense of coherence, personal control, occupational competence, and background factors such as age and socioeconomic status did not predict return to work. These results indicate that the length of rehabilitation period does matter in a dose response manner and that eight weeks of therapy may be insufficient for most participants, which is consistent with SET.

### 4.2. Psycho-Evolutionary Theory

Recently, Shanahan et al. [70] reported a study of the importance of nature experiences for urban residents. The result suggested that the health effect of nature experiences depended on the dose: a higher dose of natural experiences leads to reduced risks of high blood pressure levels as well as depression. Ottosson and Grahn [71] conducted a study examining the role of natural settings in crisis rehabilitation, and did also find a dose-effect. Their findings suggested that individuals who had many experiences of nature were less affected by their crisis than were those who had few such experiences. The symptoms in these studies indicate stress, and the results suggest that the effects may be due to a reduction in stress levels, as advocated by the Psycho-Evolutionary Theory (PET) [27]. Several recent studies also support PET, showing that stays in natural environments lead to a recovery from an onset of stress [11,72,73]. Being in a natural environment such as the Alnarp Rehabilitation Garden, could, according to PET, result in the participants obtaining lower stress levels.

Already in the 1990s, studies showed that high stress levels swiftly, within minutes, decreased when subjects experienced natural environments with restorative qualities [74]. This would indicate that individuals do not have to stay long in natural environments to obtain lower stress levels. However, a natural environment where participants can recover from high levels of stress may be a prime prerequisite for other mechanisms to operate so the participants may return to work.

### 4.3. Attention Restoration Theory

The results show that the effect size increased the longer the rehabilitation program lasted as regards return to work and occupational competence (OSA). A vital part of the OSA protocol is the ability to focus attention, be able to plan, implement what has been planned to and decided to do, and maintain the structure of everyday life; that is, abilities associated with individuals’ level of executive function. Executive function is used, for example, to focus attention, inhibit distractions, prioritize and organize information, plan everyday life, and carry out what was planned [25]. A sufficiently high level of executive function is needed in order to be able to work, study, care for your home, and maintain the structure of everyday life, while a low level of executive function is a risk factor for disease, especially mental disorders [25,75,76].

The “Attention Restoration Theory” claims that human beings effectively can recover their ability to focus attention in natural environments [24]. Several studies support this theory, showing that stays in natural environments positively affect recovery from directed attention fatigue. In addition, a higher capacity for directed attention is clearly associated with improved executive function [9,77,78,79]. The results from the study show that the participants’ level of occupational competence increases with a longer rehabilitation. Although the OSA protocol does not specifically measure the level of participants’ executive function, several variables are clearly associated with this capacity. It is hence likely that participants’ capacity of directed attention increases when staying in Alnarp Rehabilitation Garden. However, studies show that the capacity of directed attention increases relatively quickly (after about an hour) when staying in restorative natural areas [80,81]. Consequently, the possibility of increasing the capacity of directed attention in a restorative environment may also be a prerequisite for other mechanisms to work, which could result in the participants eventually returning to work.

### 4.4. The Supportive Environment Theory

When the garden was laid out, the goals were to increase the participants’ occupational competence and opportunities to return to work. This was to be achieved by carrying out activities in a natural/garden environment together with therapists and a limited number of participants who were in a similar situation [28,33]. According to SET, physical capacity as well as coping skills and executive function should increase over time. Stephen Kaplan [82] claims that in order for a garden or natural area to be supportive, the area needs to be compatible. The SET also requires that the rehabilitation site be compatible to fully support the participant. Ideally, it should contain a number of garden spaces with different types of supportive, physical characteristics, and there should be a multimodal team that may suggest meaningful activities [28,33,83].

The results from this study show that the effect size increased the longer the rehabilitation program lasted as regards return to work and occupational competence (OSA). Self-assessed abilities in the OSA protocol deal with physical energy and capacity, executive functions, as well as with being able to socialize with other people and other coping capabilities. The result could be interpreted as follows: by staying regularly in Alnarp Rehabilitation Garden, participants can lower stress levels and increase their capacity of directed attention over a period of time. With the activities carried out in this natural/garden environment, the physical strength and fitness of the participants can be developed. Executive function is adversely affected by stress, lack of sleep, sadness, loneliness, and/or lack of physical exercise [25]. Consequently, over time, participants' executive function and coping capabilities can be developed. Altogether, this leads to increased opportunities for returning to work.

A number of studies show that nature-based interventions have a positive impact on stress-related mental health and/or depression [20,21,22,23,35,40,41]. Based on deep interviews and observations [34,37,83,84], studies indicate that participants gradually develop abilities, such as increased physical strength and fitness, better sleep, and above all, that they develop new tools and strategies for coping with everyday life situations. Moreover, studies have shown measurable positive changes in participants' self-perceived experiences of everyday activities [36]. The above results indicate that when participants, during a period of time and without stress, are given the opportunity to think and reflect on their situation, they can develop new tools and strategies, as well as practice and try them to cope with their daily lives. This entails increased opportunities to return to work. Still, this takes time, and eight weeks might not be sufficient to gain these skills. In the regression analysis, the length of the rehabilitation program was found to be the significant and decisive factor in predicting return to work.

Over 75% of those who received care at Alnarp had university educations, with the majority working in the health care or education sectors as nurses, teachers, psychologists, medical doctors, etc. These professions all require emotional commitment, which has proved to involve a higher risk of burnout and exhaustion [85]. As such, the participants’ occupations were those that might be expected for an intervention group severely affected by exhaustion and burnout. Across the world, problems with burnout and exhaustion increase. Nature-based rehabilitation may provide opportunities to return to work, especially if the participants are offered sufficiently long rehabilitation.

### 4.5. Limitations

The study was not randomized, which is a limitation. In 1991, however, Andrews pointed out that the randomized controlled trial design is challenging to use in rehabilitation research, and that other strategies can provide equally safe or safer results [86]. Graham et al. [87] propose smaller studies with a stronger focus on individual participants as a good research strategy for developing evidence-based solutions. Because they have many advantages, it has been recommended that natural and quasi-natural experiments be used more often in rehabilitation research. Among other things, ethical problems are reduced, and the result gives a fairer picture of what real rehabilitation looks like [43,88]. The scientific method we used in this study was quasi-experimental [46] with an intention to treat [47]. No participants exited the intervention, but some ended their participation earlier than planned, and some stayed longer than planned.

The response rate on distributed forms was high. In general, the forms were returned to us. The study included 106 participants, and only 11 did not reply at one year follow-up. The self-assessment forms we received were completely or almost completely filled in. The number of participants in the Tables varies somewhat depending on which self-assessment protocol is reported. Using imputations would have increased the response rate, but we chose not to do this.

The rehabilitation which is described in this article is not too different from horticultural therapy in the United States, UK, Germany, Japan, Korea, and several other countries. In Materials and Methods, the reader can find references that in great detail describe activities, programs, and the design of the garden. From this, others can carry out similar activities. One strength of this study is that the three groups were similar in terms of gender, marital status, age, socioeconomic status, primary diagnosis, length of illness, and baseline values for outcome measures.

## 5. Conclusions

The outcomes from this study indicate that nature-based rehabilitation increases the likelihood of returning to work. In order to achieve a strong effect, however, the results suggest that the rehabilitation needs to be carried out over a longer period of time.

## Figures and Tables

**Table 1:** Baseline characteristics for the patients participating in the different rehabilitation periods, (*n* = 106).

	8 Weeks	12 Weeks	24 Weeks
*n*	44	48	14
Mean period (range)	7.8 (4.0–9.4)	13.4 (10.0–17.0)	23.2 (18.0–29.8)
Mean age	46.3	45.7	44.1
Sex F/M	39/5	43/5	14/0
Marital status: Married or living with another adult/Single	30/14	32/16	8/6
Socioeconomic status	2.82	2.61	3.22
Time on sick leave (year)	3.68	3.71	3.82
Primary Diagnosis F43/F32	30/14	34/14	13/1
Additional consultation (%)	8 (18)	10 (21)	3 (21)

**Table 2:** Return to work 12 months after start of rehabilitation (*n* = 95).

Percent	Paid Work (%)	Job Training (%)	Customized Job Training (%)	Combined (Paid Work, Job Training, and Customized Job Training) (%)
0	53 (55.8)	78 (82.1)	89 (93.7)	30 (31.6)
25	8 (8.4)	7 (7.3)	1 (1.0)	16 (16.8)
50	11 (11.6)	5 (5.3)	3 (3.2)	19 (20.0)
75	9 (9.5)	0 (0.0)	0 (0.0)	9 (9.5)
100	14 (14.7)	5 (5.3)	2 (2.1)	21 (22.1)

**Table 3:** Outcome measures for the patients participating in the different rehabilitation periods. Mean values at baseline and follow-up regarding return to work (RTW, %), occupational competence (OSA), personal control (Mastery), and sense of coherence (SoC). Paired *t*-test, Hedges’ g effect size (ES); with magnitude high (H), medium (M) or small (S) (*n* = 106).

Rehabilitation Period	8 Weeks						12 Weeks						24 Weeks					
Variable	*n*	Mean Start–End	Diff	ES (Magn.)	*t*	*p*	*n*	Mean Start–End	Diff	ES (Magn.)	*t*	*p*	*n*	Mean Start–End	Diff	ES (Magn.)	*t*	*p*
RTW	40	0–20.0	20.0	0.85 (H)	3.77	<0.001	43	0.0–34.9	34.9	1.22 (H)	5.65	<0.0001	12	0.0–43.8	43.8	1.49 (H)	3.66	<0.01
OSA	34	36.5–41.5	5.0	0.64 (M)	4.22	<0.001	37	36.8–44.9	8.1	1.07 (H)	6.23	<0.0001	10	35.4–44.9	9.5	1.46 (H)	3.34	<0.01
Mastery	36	17.0–20.0	3.0	0.67 (M)	4.94	<0.0001	40	17.5–20.7	3.2	0.70 (M)	4.98	<0.0001	12	18.5–21.7	3.2	0.68 (M)	3.01	=0.01
SoC	34	47.2–54.8	7.6	0.60 (M)	3.84	<0.001	37	46.7–59.7	13.0	1.10 (H)	6.28	<0.0001	10	49.8–59.1	9.3	0.56 (M)	2.67	<0.05

## References

[1] Salomon J.A., Wang H., Freeman M.K., Vos T., Flaxman A.D., Lopez A.S., Murray C.J.L. (2012). Healthy life expectancy for 187 countries, 1990–2010: A systematic analysis for the Global Burden Disease Study 2010. Lancet.

[2] Steel Z., Marnane C., Iranpour C., Chey T., Jackson J.W., Patel V., Silove D. (2014). The global prevalence of common mental disorders: A systematic review and meta-analysis. 1980–2013. Int. J. Epidemiol..

[3] Försäkringskassan (2014). Sjukfrånvaro i Psykiska Diagnoser En Studie av Sveriges Befolkning 16–64 år.

[4] Slany C., Schutte S., Chastang J.-F., Parent-Thirion A., Vermeylen G., Niedhammer I. (2014). Psychosocial work factors and long sickness absence in Europe. Int. J. Environ. Res. Public Health.

[5] Kuoppala J., Lamminpää A. (2008). Rehabilitation and work ability: A systematic literature review. J. Rehabil. Med..

[6] Swedish Government (2011). Rehabiliteringsrådets Slutbetänkande [Final Commission Report from the Rehabilitation Council].

[7] Vingård E. (2015). Psykisk Ohälsa, Arbetsliv och Sjukfrånvaro. En Kunskapsöversikt och Rapport. http://forte.se/wp-content/uploads/2015/04/psykisk-ohalsa-arbetsliv.pdf.

[8] SBU (2014). Arbetsmiljöns Betydelse för Symtom på Depression och Utmattningssyndrom, En Systematisk Litteraturöversikt [The Importance of the Working Environment for Symptoms of Depression and Exhaustion, A Systematic Literature Review], SBU-Rapport nr 223.

[9] Bratman G.N., Hamilson J.P., Daily G.C. (2015). The impacts of nature experience on human cognitive function and mental health. Landsc. Urban Plan..

[10] James P., Hart J.E., Banay R.F., Laden F. (2016). Exposure to greenness and mortality in a nationwide prospective cohort study of women. Environ. Health Perspect..

[11] Jiang B., Chang C.-Y., Sullivan W.C. (2014). A dose of nature: Tree cover, stress reduction, and gender differences. Landsc. Urban Plan..

[12] Li Q. (2010). Effect of forest bathing trips on human immune function. Environ. Health Prev. Med..

[13] Ward Thompson C., Roe J., Aspinall P., Mitchell R., Clow A., Miller D. (2012). More green space is linked to less stress in deprived communities: Evidence from salivary cortisol patterns. Landsc. Urban Plan..

[14] Cetin M., Adiguzel F., Kaya O., Sahap A. (2016). Mapping of bioclimatic comfort for potential planning using GIS in Aydin. Environ. Dev. Sustain..

[15] Stigsdotter U.K., Palsdottir A.M., Burls A., Chermaz A., Ferrini F., Grahn P., Nilsson K., Sangster M., Gallis C., Hartig T., de Vries S., Seeland K. (2011). Nature-based therapeutic interventions. Forest, Trees and Human Health.

[16] Lidwall U., Olsson-Bohlin C. (2017). Lång väg tillbaka till arbete vid sjukskrivning, Psykiatriska diagnose. [Long way back to work when on sick leave, psychiatric diagnoses]. Korta Analyser, Försäkringskassan.

[17] Tyson M.M. (2007). The Healing Landscape: Therapeutic Outdoor Environment.

[18] Annerstedt M., Währborg P. (2011). Nature assisted therapy: Systematic review of controlled and observational studies. Scand. J. Public Health.

[19] Blake M., Mitchell G. (2016). Horticultural therapy in dementia care: A literature review. Nurs. Stand..

[20] Cipriani J., Benz A., Holmgren A., Kinter D., McGarry J., Rufino G. (2017). A systematic review of the effects of horticultural therapy on persons with mental health conditions. Occup. Ther. Ment. Health.

[21] Clatworthy J., Hinds J., Camic P.M. (2013). Gardening as a mental health intervention: A review. Ment. Health Rev..

[22] Jang E.J., Han G.W., Hong J.W., Yoon S.E., Pak C.H. (2010). Meta-analysis of research papers on horticultural therapy program effect. Korean J. Horticult. Sci. Technol..

[23] Kim J.H., Kwon S.B., Kim H.J., Choi G.H., Lee H.M. (2016). Effects of horticultural therapy for the Korean elderly: A systematic literature review. J. Korean Biol. Nurs. Sci..

[24] Kaplan S. (2001). Meditation, restoration, and the management of mental fatigue. Environ. Behav..

[25] Diamond A. (2013). Executive functions. Annu. Rev. Psychol..

[26] Kaplan S., Berman M.G. (2010). Directed attention as a common resource for executive functioning and self-regulation. Perspect. Psychol. Sci..

[27] Ulrich R.S., Kellert S.R., Wilson E.O. (1993). Biophilia, biophobia, and natural landscapes. The Biophilia Hypothesis.

[28] Grahn P., Tenngart Ivarsson C., Stigsdotter U.K., Bengtsson I.-L., Ward Thompson C., Aspinal P., Bell S. (2010). Using affordances as a health promoting tool in a therapeutic garden. Innovative Approaches to Researching Landscape and Health.

[29] Melchert T.P. (2015). Biopsychosocial Practice.

[30] Grahn P., Mossberg F. (2011). Om stödjande miljöer och rofyllda ljud. [On supportive environments and serene sounds]. Ljudmiljö, Hälsa Och Stadsbyggnad.

[31] Adevi A.A. (2012). Supportive Nature—And Stress. Wellbeing in Connection to Our Inner and Outer Landscape.

[32] Pálsdóttir A.M. (2014). The Role of Nature in Rehabilitation for Individuals with Stress-Related Mental Disorders. Alnarp. Rehabilitation Garden as Supportive Environment.

[33] Stigsdotter U., Grahn P. (2003). Experiencing a garden: A healing garden for people suffering from burnout diseases. J. Ther. Hort..

[34] Pálsdóttir A.M., Persson D., Persson B., Grahn P. (2014). The journey of recovery and empowerment embraced by nature—Clients’ perspectives on nature-based rehabilitation in relation to the role of the natural environment. Int. J. Environ. Res. Public Health.

[35] Gonzalez M.T., Hartig T., Patil G.G., Martinsen E.W., Kirkevold M. (2009). Therapeutic horticulture in clinical depression: A prospective study. Res. Theory Nurs. Pract..

[36] Pálsdóttir A.M., Grahn P., Persson D. (2014). Changes in experienced value of everyday occupations after nature-based vocational rehabilitation. Scand. J. Occup. Ther..

[37] Poulsen D.V., Stigsdotter U.K., Djernis D., Sidenius U. (2016). “Everything just seems much more right in nature”: How veterans with post-traumatic stress disorder experience nature-based activities in a forest therapy garden. Health Psychol. Open.

[38] Sahlin E., Ahlborg G., Tenenbaum A., Grahn P. (2015). Using nature-based rehabilitation to restart a stalled process of rehabilitation in individuals with stress-related mental illness. Int. J. Environ. Res. Public Health.

[39] Währborg P., Petersson I.F., Grahn P. (2014). Nature-assisted rehabilitation for reactions to severe stress and/or depression in a rehabilitation garden: Long-term follow-up including comparisons with a matched population-based reference cohort. J. Rehab. Med..

[40] Kam M.C.Y., Siu A.M.H. (2010). Evaluation of a horticultural activity programme for persons with psychiatric illness. Hong Kong J. Occup. Ther..

[41] Nordh H., Grahn P., Währborg P. (2009). Meaningful activities in the forest, a way back from exhaustion and long-term sick leave. Urban For. Urban Green..

[42] Nyman P. Experimentell Design Inom Samhällsvetenskapen. http://www.parnyman.com/files/texts/experiment.pdf.

[43] Prvu Bettger J.A., Stineman M.G. (2007). Effectiveness of multidisciplinary rehabilitation services in postacute care: State-of-the-science. A review. Arch. Phys. Med. Rehabil..

[44] Craig P., Cooper C., Gunnell D., Haw S., Lawson K., Macintyre S., Ogilvie D., Petticrew M., Reeves B., Sutton M. (2012). Using natural experiments to evaluate population health interventions: New Medical Research Council guidance. J. Epidemiol. Community Health.

[45] Hallberg K., Eno J., Wright J. (2015). Quasi-Experimental Designs. International Encyclopedia of the Social & Behavioral Sciences.

[46] Rockers P.C., Røttingen J.-A., Shemilt I., Tugwell P., Bärnighausen T. (2015). Inclusion of quasi-experimental studies in systematic reviews of health systems research. Health Policy.

[47] Montori V.M., Guyatt G.H. (2001). Intention-to-treat principle. CMAJ.

[48] World Health Organization (2011). ICD-10. International Statistical Classification of Diseases and Related Health Problems, 10th Revision.

[49] Stigsdotter U., Grahn P. (2002). What makes a garden a healing garden?. J. Ther. Hortic..

[50] Orians G.H., Penning-Rowsell E.C., Lowenthal D. (1986). An ecological and evolutionary approach to landscape aesthetics. Landscape Meanings and Values.

[51] Appleton J. (1975). The Experience of Landscape.

[52] Coss R.G. (1990). All that glistens: Water connotations in surface finishes. Ecol. Psychol..

[53] Tenngart Ivarsson C., Hägerhäll C.M. (2008). The perceived restorativeness of gardens—Assessing the restorativeness of a mixed built and natural scene type. Urban For. Urban Green..

[54] Grahn P., Stigsdotter U.K. (2010). The relation between perceived sensory dimensions of urban green space and stress restoration. Landsc. Urban Plan..

[55] Kielhofner G. (2002). A Model of Human Occupation: Theory and Application.

[56] Kielhofner G., Forsyth K. (2001). Measurement properties of a client self-report for treatment planning and documenting occupational therapy outcomes. Scand. J. Occup. Ther..

[57] Pearlin L.I., Schooler C. (1978). The structure of coping. J. Health Soc. Behav..

[58] Pearlin L.I., Menaghan E.G., Lieberman M.A., Mullen J.T. (1981). The stress process. J. Health Soc. Behav..

[59] Bengtsson-Tops A. (2004). Mastery in patients with schizophrenia living in the community. J. Psychiatr. Ment. Health Nurs..

[60] Boscarino J.A., Figley C.R., Adams R.E. (2004). Compassion fatigue following the September 11 terrorist attacks: A study of secondary trauma among New York City social workers. Int. J. Emerg. Ment. Health.

[61] Stephens T., Dulberg C., Joubert N. (2000). Mental health of the Canadian population: A comprehensive analysis. Chronic Dis. Can..

[62] Nyqvist F., Forsman A.K., Cattan M. (2013). A comparison of older workers’ and retired older people’s social capital and sense of mastery. Scand. J. Public Health.

[63] Eriksson M., Lindström B. (2005). Validity of Antonovsky’s sense of coherence scale: A systematic review. J. Epidemiol. Community Health.

[64] Ahola A.J., Saraheimo M., Forsblom C., Hietala K., Groop P.-H. (2010). The cross-sectional associations between sense of coherence and diabetic microvascular complications, glycaemic control, and patients’ conceptions of type 1 diabetes. Health Qual. Life Outcomes.

[65] Miles J., Shevlin M. (2001). Applying Regression and Correlation.

[66] Norman G.R., Streiner D.L. (2008). Biostatistics: The Bare Essentials.

[67] Cohen J. (1988). Statistical Power Analysis for the Behavioral Sciences.

[68] Hedges L.V. (1981). Distribution theory for Glass’ estimator of effect size and related estimators. J. Educ. Stat..

[69] Sullivan G.M., Feinn R. (2012). Using effect size—Or why the *p* value is not enough. J. Grad Med. Educ..

[70] Shanahan D.F., Bush R., Gaston K.J., Lin B.B., Dean J., Barber E., Fuller R.A. (2016). Health benefits from nature experiences depend on dose. Sci. Rep..

[71] Ottosson J., Grahn P. (2008). The role of natural settings in crisis rehabilitation. Landsc. Res..

[72] Beil K., Hanes D. (2013). The influence of urban natural and built environments on physiological and psychological measures of stress. Int. J. Environ. Res. Public Health.

[73] Berto R. (2014). The role of nature in coping with psycho-physiological stress: A literature review on restorativeness. Behav. Sci..

[74] Ulrich R.S., Simons R.F., Losito B.D., Fiorito E., Miles M.A., Zelson M. (1991). Stress recovery during exposure to natural and urban environments. J. Environ. Psychol..

[75] Bailey C.E. (2007). Cognitive accuracy and intelligent executive function in the brain and in business. Ann. N. Y. Acad. Sci..

[76] Duncan G.J., Dowsett C.J., Claessens A., Magnuson K., Huston A.C., Klebanov P., Pagani L.S., Feinstein L., Engel M., Brooks-Gunn J. (2007). School readiness and later achievement. Dev. Psychol..

[77] Berman M.G., Kross E., Krpan K.M., Askren M.K., Burson A., Deldin P.J., Kaplan S., Sherdell L., Gotlib I.H., Jonides J. (2012). Interacting with nature improves cognition and affect for individuals with depression. J. Affect. Disord..

[78] Lee K.E., Williams K.J.H., Sargent L.D., Williams N.S.G., Johnson K.A. (2015). 40-second green roof views sustain attention: The role of micro-breaks in attention restoration. J. Environ. Psychol..

[79] Ohly H., White M.P., Wheeler B.W., Bethel A., Ukoumunne O.C., Nikolaou V., Garside R. (2016). Attention restoration theory: A systematic review of the attention restoration potential of exposure to natural environments. J. Toxicol. Environ. Health B.

[80] Berman M.G., Jonides J., Kaplan S. (2008). The cognitive benefits of interacting with nature. Psychol. Sci..

[81] Ottosson J., Grahn P. (2005). A comparison of leisure time spent in a garden with leisure time spent indoors: On measures of restoration in residents in geriatric care. Landsc. Res..

[82] Kaplan S., Sorte G.J. (1990). Parks for the future–A psychologist view. Parks for the Future.

[83] Grahn P., Ottosson Å (2010). Trädgårdsterapi Alnarpsmetoden: Att Ta Hjälp Av Naturen Vid Stress Och Utmattning.

[84] Sahlin E., Matuszczyk J.V., Ahlborg G., Grahn P. (2012). How do participants in nature-based therapy experience and evaluate their rehabilitation?. J. Ther. Hortic..

[85] Schaufeli W.B., Leiter M.P., Maslach C. (2009). Burnout: 35 years of research and practice. Career Dev. Int..

[86] Andrews K. (1991). The limitations of randomized controlled trials in rehabilitation research. Clin. Rehabil..

[87] Graham J.E., Karmarkar A.M., Ottenbacher K.J. (2012). Small sample research designs for evidence-based rehabilitation: Issues and methods. Arch. Phys. Med. Rehabil..

[88] Hart T., Whyte J., Poulsen I., Spangsberg Kristensen K., Nordenbo A.M., Chervoneva I., Vaccaro M.J. (2016). How do intensity and duration of rehabilitation services affect outcomes from severe traumatic brain injury? A natural experiment comparing health care delivery systems in 2 developed nations. Arch. Phys. Med. Rehabil..

